# The Inflammasome Components NLRP3 and ASC Act in Concert with IRGM To Rearrange the Golgi Apparatus during Hepatitis C Virus Infection

**DOI:** 10.1128/JVI.00826-20

**Published:** 2021-01-13

**Authors:** Coralie F. Daussy, Sarah C. Monard, Coralie Guy, Sara Muñoz-González, Maxime Chazal, Marit W. Anthonsen, Nolwenn Jouvenet, Thomas Henry, Marlène Dreux, Eliane F. Meurs, Marianne Doré Hansen

**Affiliations:** a CNRS UMR 3569, Hepacivirus and Innate Immunity, Institut Pasteur, Paris, France; b École Normale Supérieure de Lyon, Lyon, France; c Université Claude Bernard Lyon I, Lyon, France; d Université de Lyon, Lyon, France; e CIRI, INSERM, U1111, Université Claude Bernard Lyon 1, Lyon, France; f CNRS, UMR5308, École Normale Supérieure de Lyon, Lyon, France; g Université de Lyon, Lyon, France; h CNRS UMR 3569, Virology Department, Institut Pasteur, Paris, France; i Department of Clinical and Molecular Medicine, Faculty of Medicine and Health Sciences, Norwegian University of Science and Technology, Trondheim, Norway; j Clinic of Laboratory Medicine, Department of Medical Microbiology, St. Olavs Hospital, Trondheim, Norway; University of Southern California

**Keywords:** ASC, Golgi rearrangement, HCV, IRGM, NLRP3, ZIKV

## Abstract

Hepatitis C virus (HCV) infection triggers Golgi fragmentation through the Golgi-resident protein immunity-related GTPase M (IRGM). Here, we report the roles of NLRP3 (NOD-, LRR- and pyrin domain-containing protein 3) and ASC (apoptosis-associated speck-like protein containing a caspase activation and recruitment domain [CARD]), two inflammasome components, in the initial events leading to this fragmentation. We show that ASC resides at the Golgi with IRGM at homeostasis. Upon infection, ASC dissociates from both IRGM and the Golgi and associates with HCV-induced NLRP3. NLRP3 silencing inhibits Golgi fragmentation. ASC silencing disrupts the Golgi structure in both control and infected cells and reduces the localization of IRGM at the Golgi. IRGM depletion in the ASC-silenced cells cannot totally restore the Golgi structure. These data highlight a role for ASC, upstream of the formation of the inflammasome, in regulating IRGM through its control on the Golgi. A similar mechanism occurs in response to nigericin treatment, but not in cells infected with another member of the *Flaviviridae* family, Zika virus (ZIKV). We propose a model for a newly ascribed function of the inflammasome components in Golgi structural remodeling during certain stimuli.

**IMPORTANCE** Numerous pathogens can affect cellular homeostasis and organelle dynamics. Hepatitis C virus (HCV) triggers Golgi fragmentation through the immunity-related GTPase M (IRGM), a resident Golgi protein, to enhance its lipid supply for replication. Here, we reveal the role of the inflammasome components NLRP3 and ASC in this process, thus uncovering a new interplay between effectors of inflammation and viral infection or stress. We show that the inflammasome component ASC resides at the Golgi under homeostasis and associates with IRGM. Upon HCV infection, ASC is recruited to NLRP3 and dissociates from IRGM, causing Golgi fragmentation. Our results uncover that aside from their known function in the inflammation response, these host defense regulators also ensure the maintenance of intact intracellular structure in homeostasis, while their activation relieves factors leading to Golgi remodeling.

## Abstract (precis)

Numerous pathogens can affect cellular homeostasis and organelle dynamics. Hepatitis C virus (HCV) triggers Golgi fragmentation through the immunity-related GTPase M (IRGM), a resident Golgi protein, to enhance its lipid supply for replication.

## INTRODUCTION

Hepatitis C virus (HCV) is a major causative agent of chronic hepatitis and hepatocellular carcinoma worldwide. Twenty percent of HCV-infected individuals can eliminate this virus, but chronic infection occurs in 80% of cases. In the absence of efficient treatment, such as direct antiviral agent (DAA) therapy (1), there is progressive degradation of the liver through inflammation, fibrosis, cirrhosis, and hepatocarcinoma (2). Chronic HCV infection will remain a major global health problem in decades to come. HCV chronic infection is recognized to promote the dysregulation of lipid metabolism and thereby can participate in many ways in the genesis of steatosis (3). Similar to all positive-strand RNA viruses studied thus far (4), HCV replicates its genome in association with intracellular membrane rearrangements. For HCV, the replication takes place within a so-called membranous web (MW) derived from the endoplasmic reticulum (ER). The HCV MW has a complex morphology consisting of clusters of single-, double-, and multimembrane vesicles, which likely include autophagosomes, and is present at proximity to the lipid droplets (5, 6). The formation of MW is regulated by distinct HCV nonstructural (NS) proteins through sequential interactions with several host factors (5, 7). For example, membrane-associated NS5A may bind directly to phosphatidylinositol-4 kinase III alpha (PI4KIIIα) to initiate morphogenesis of viral replication sites (7). Numerous other cellular components are subverted by HCV to promote MW formation. In addition, the HCV life cycle is also closely tied to lipid metabolism in infected cells (8).

We have recently reported that HCV exploits the immunity-related GTPase M (IRGM), previously identified as a risk factor for Crohn’s disease and tuberculosis and for its role in autophagy (9) to regulate the activity of the vesicular transport protein GBF1 and of the small Arf1 GTPase (10), thereby leading to Golgi fragmentation and facilitating HCV replication through lipid supply (11). However, the initial events leading to the activation of IRGM upon HCV infection are not known.

The NLRP3 inflammasome is a multiprotein cytosolic complex that can be activated by diverse stimuli: e.g., RNA viruses, endogenous danger signals, and environmental irritants. It results in its assembly causing activation of caspase-1 and secretion of the proinflammatory cytokines interleukin-1β (IL-1β) and IL-18 (12–15). In response to infection of the liver with hepatitis C virus, activation of the NLRP3 inflammasome has been clearly related to the role of the liver-resident macrophages surrounding the hepatocytes (16–18). However, a direct role of the hepatocytes can also be recognized, in response to either HCV infection (19) or activation of stress (20, 21). In particular, the activation of the NLRP3 inflammasome in cultured hepatoma cells upon HCV infection can ultimately lead to the caspase-1-mediated form of programmed cell death called pyroptosis (19). Whatever the nature of the cells, activation of the NLRP3 inflammasome requires association of NLRP3 with caspase-1 through ASC (apoptosis-associated speck-like protein containing a caspase activation and recruitment domain [CARD]) (22).

IRGM contains a globular N-terminal GTPase domain and, like ASC, a C-terminal helical CARD. Here we investigated the relationship between the inflammasome components and IRGM, with regard to Golgi structure during viral infection (i.e., HCV and Zika virus [ZIKV]) or chemical stimuli culminating in the assignment of a newly defined relationship between components of the NLRP3 inflammasome and host intracellular compartments in response to HCV infection or some stimuli or stress.

## RESULTS

### HCV infection induces NLRP3 inflammasome components.

**FIG 1:**
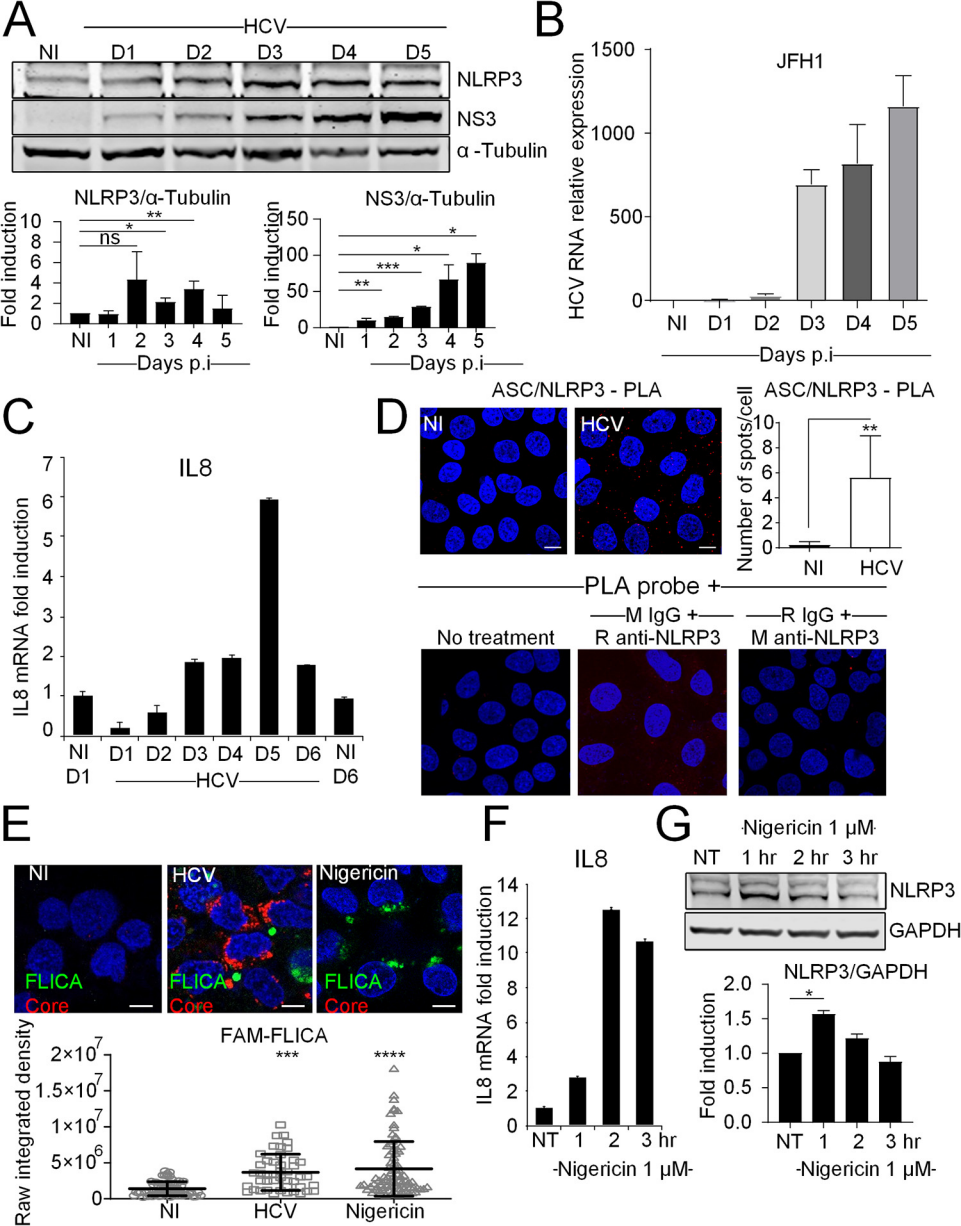
HCV infection induces NLRP3 inflammasome components. (A) The kinetics of expression of NLRP3 and HCV NS3 protein in uninfected (NI) and HCV-infected Huh7 cells were examined by immunoblotting. A blot from one representative experiment is shown. NLRP3 and HCV NS3 protein levels were normalized to α-tubulin and visualized as means ± SD (*n* = 3 independent experiments). (B) HCV RNA levels were determined by RT-qPCR in cells infected with HCV. (C) IL-8 mRNA levels were determined by RT-qPCR in Huh7 cells infected with HCV. (D) Huh7 cells were infected with HCV for 5 days. The proximity ligation assay (PLA) was performed with antibodies directed against ASC and NLRP3, with quantification of the number of PLA dots. The data shown are means ± SD (*n* = 3 independent experiments with >180 cells per condition). Negative controls for PLA are indicated as M (mouse) and R (rabbit). (E) Huh7 cells were either infected with HCV (5 days) or treated with nigericin (1 μM, 3 h of treatment). Control cells were left uninfected and untreated. Cells were incubated for 1 h with the FLICA reagent (which stains the active form of caspase-1 green) prior to fixation. Fixed cells were immunostained with antibodies against HCV core protein (red), and nuclei were stained with DAPI (blue). The raw integrated density was calculated for FLICA staining. (F) IL-8 mRNA levels were determined by RT-qPCR in Huh7 cells treated with 1 μM nigericin for the indicated time points. (G) Representative image of immunoblotting of NLRP3 in nigericin-treated cells. The data shown are means ± SD (*n* = 3 independent experiments). Scale bar, 5 μm. ns, not significant. *, *P* < 0.05; **, *P* < 0.01; ***, *P* < 0.001; ****, *P* < 0.0001.

Activation of the NLRP3 inflammasome first requires increased expression of NLRP3, or priming, which can occur through NF-κB activation (23). Here, we have examined the induction of NLRP3 by HCV in Huh7 hepatoma cells and found NLRP3 protein levels to be induced by HCV infection starting from day 1 postinfection and increasing significantly in association with augmented levels of HCV NS3 protein levels and HCV RNA (Fig. 1A and B), as well as induction of IL-8, used as a marker of NF-κB activation (24) (Fig. 1C). Furthermore, we observed an induction of the interaction between NLRP3 and ASC upon HCV infection, as measured by the proximity ligation assay (PLA) (Fig. 1D), but not in control cells. Activation of caspase-1 in response to HCV infection was demonstrated using a fluorescent reporter of caspase-1 substrate (FAM-FLICA) and by comparison with a 3-h treatment with nigericin, a well-known activator of the NLRP3 inflammasome (25) (Fig. 1E). Such a 3-h treatment with nigericin was sufficient to trigger induction of IL-8, chosen as a marker of NF-κB activation, and to induce NLRP3 expression in Huh7 cells (Fig. 1F and G).

### Golgi fragmentation upon HCV infection involves NLRP3.

**FIG 2:**
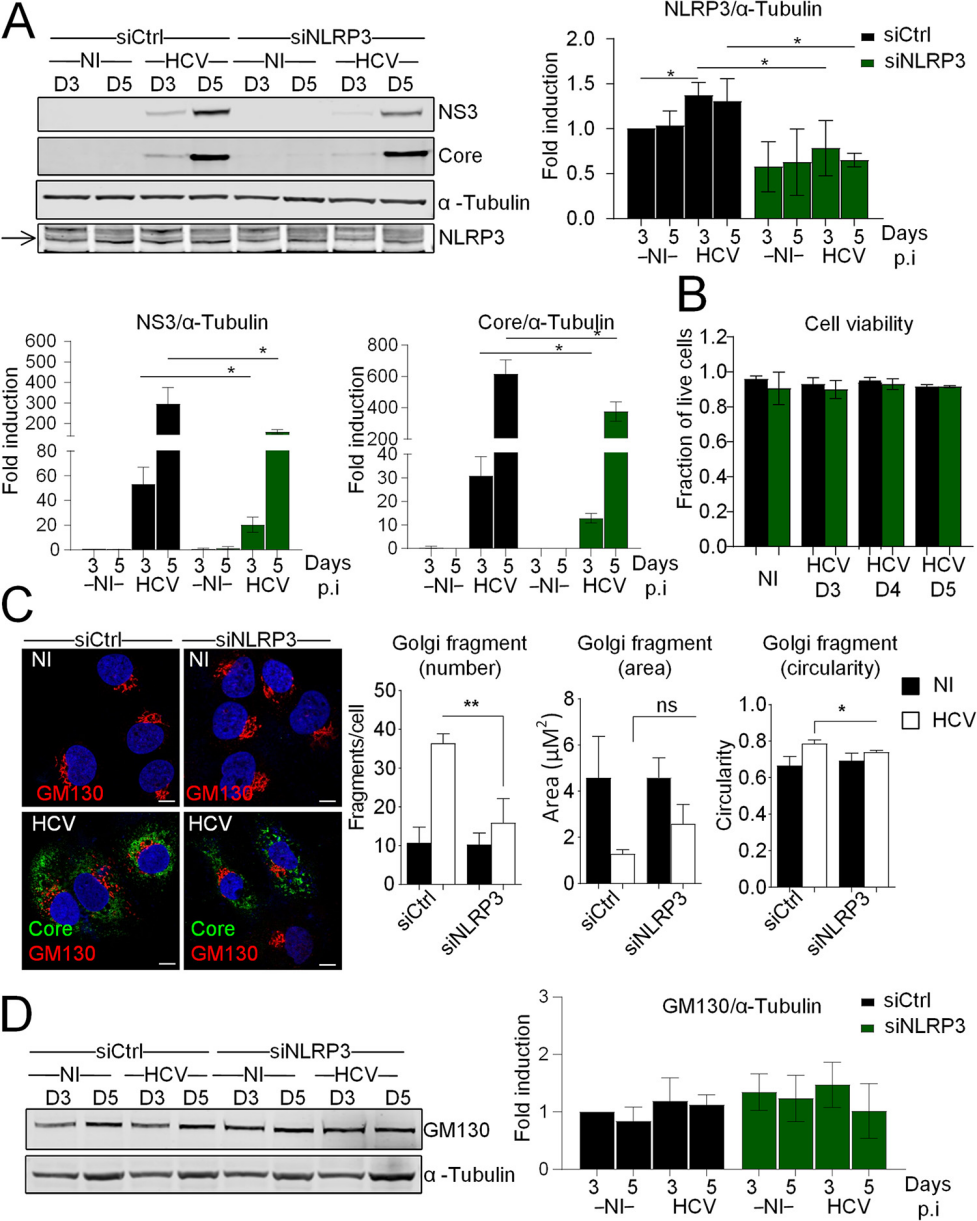
Golgi fragmentation upon HCV infection involves NLRP3. (A) Huh7 cells were reverse transfected with siRNAs against NLRP3 (siNLRP3) or control siRNA (siCtrl) prior to infection with HCV (MOI of 1) for 3 and 5 days. NLRP3 knockdown was controlled by immunoblotting, and the effect of NLRP3 depletion on HCV protein levels was examined by immunoblotting with HCV NS3 and HCV core antibodies. Protein levels were normalized to α-tubulin. A blot from one representative experiment is shown. An arrow marks the NLRP3 protein band. (B) The cell viabilities of cells transfected with siRNAs against NLRP3 or control siRNA that were infected or not with HCV for the indicated time points were examined. (C) Representative images of Huh7 cells depleted of NLRP3 or not and immunostained with antibodies against GM130 (red) and HCV core (green). DAPI staining marks nuclei (blue). The characteristics of Golgi fragments were calculated. The data shown are means ± SD (*n* = 3 independent experiments with >20 cells per condition). (D) The effect of NLRP3 knockdown on GM130 total protein levels was controlled by immunoblotting. A blot from one representative experiment is shown. The data shown are means ± SD (*n* = 3 independent experiments). Scale bar, 5 μm. ns, not significant. *, *P* < 0.05; **, *P* < 0.01.

To characterize the role of NLRP3 in HCV infection, we first studied the effect of NLRP3 depletion on HCV protein levels in Huh7 cells. Immunoblotting staining confirmed that knockdown of NLRP3 by small interfering RNA (siRNA) caused reduced expression of the expected NLRP3 band but also a significant reduction in both HCV NS3 and HCV core protein levels, suggesting that NLRP3 supports continuous HCV replication (Fig. 2A). We observed no reduction in cell viability upon NLRP3 depletion in resting cells or in HCV-infected cells (Fig. 2B). We had shown earlier that HCV triggers Golgi fragmentation to facilitate its replication through lipid supply (11). To examine whether the proviral role of NLRP3 could result in Golgi fragmentation, HCV infection was performed in cells in which NLRP3 expression was silenced prior to infection. Cells were costained with antibodies against GM130 and HCV core protein. Staining of the HCV core protein was used as a marker to identify HCV-infected cells. Confocal acquisition followed by a detailed analysis and quantification of Golgi phenotype revealed that the changes in Golgi morphology caused by HCV infection (clustered vesicles as opposed to tubulovesicular structures in the uninfected cells, as shown previously [11]) are less pronounced in the cells silenced for NLRP3 compared to the situation in the control siRNA (siCtrl)-treated cells (Fig. 2C). In addition, we found that silencing of NLRP3 did not reduce the total protein levels of GM130 (Fig. 2D), ruling out that lower levels of total GM130 protein could be the cause of the observed reduction in Golgi fragmentation. These results suggest the existence of a link between NLRP3 and the regulation of HCV-induced Golgi rearrangement.

### HCV infection triggers dissociation of ASC from IRGM at the Golgi.

**FIG 3:**
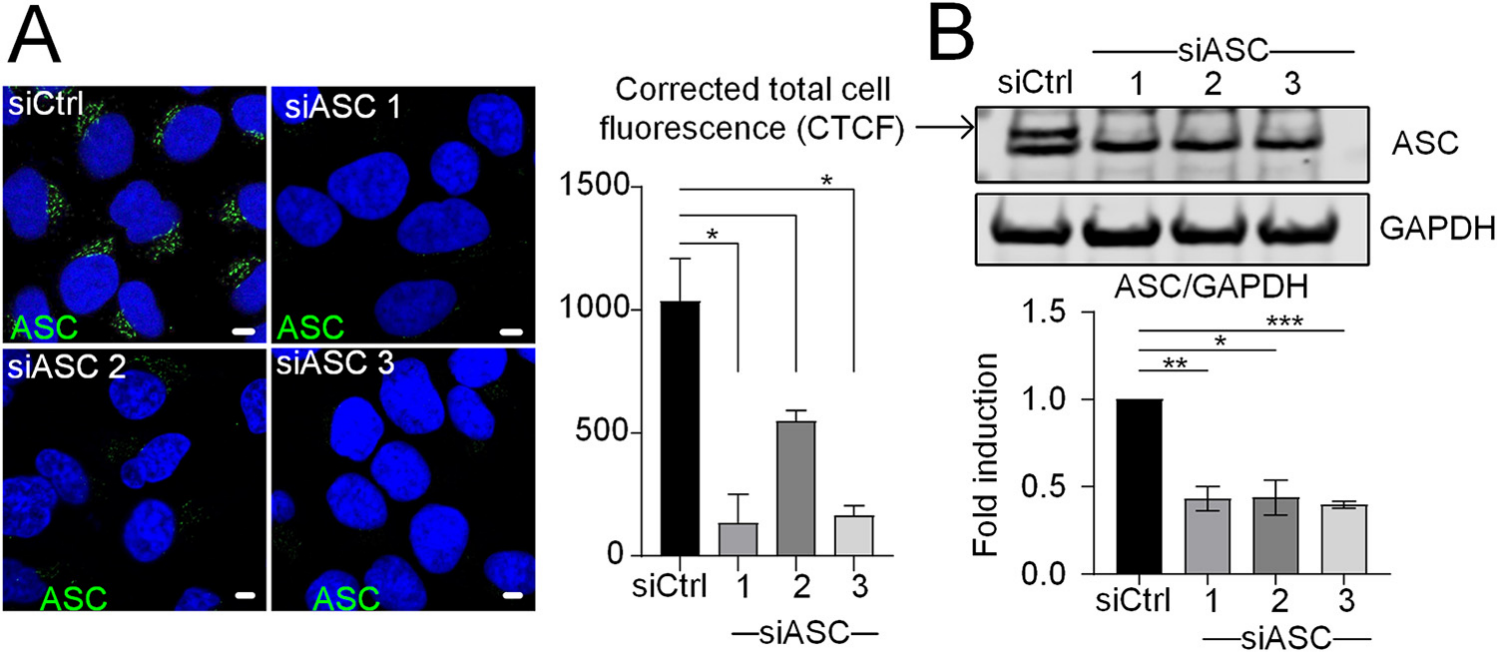
Specificity of the ASC antibodies. (A) Huh7 cells were treated with three different siRNAs against ASC or a control siRNA. (A) Intracellular staining of endogenous ASC. Cells were examined by confocal microscopy, and the corrected total cell fluorescence (CTCF) was calculated using ImageJ. DAPI staining marks nuclei (blue). (B) Total protein levels were examined by immunoblotting with ASC antibody. The arrow points out the specific ASC protein band position. The data shown are means ± SD (*n* = 3 independent experiments). Scale bar, 5 μm. *, *P* < 0.05; **, *P* < 0.01; ***, *P* < 0.001.

The formation of the NLRP3 inflammasome complex requires interaction of NLRP3 with ASC and caspase-1. Since both ASC and the immunity-related GTPase M (IRGM), which regulates the HCV-induced Golgi fragmentation (11), contain a CARD, an interaction motif regulating several processes relating to innate responses, we examined the relationship between IRGM and ASC during HCV infection by using subcellular imaging analysis. Due to some uncertainties about the cellular localization of ASC from the literature (26–28), we first silenced its expression in Huh7 cells (using three different siRNAs) before immunostaining. This resulted in a strong reduction of the expression of ASC both by immunofluorescence (Fig. 3A) and by Western blotting (Fig. 3B), thus confirming the specificity of our antibodies and the efficacy of the siRNAs.

**FIG 4:**
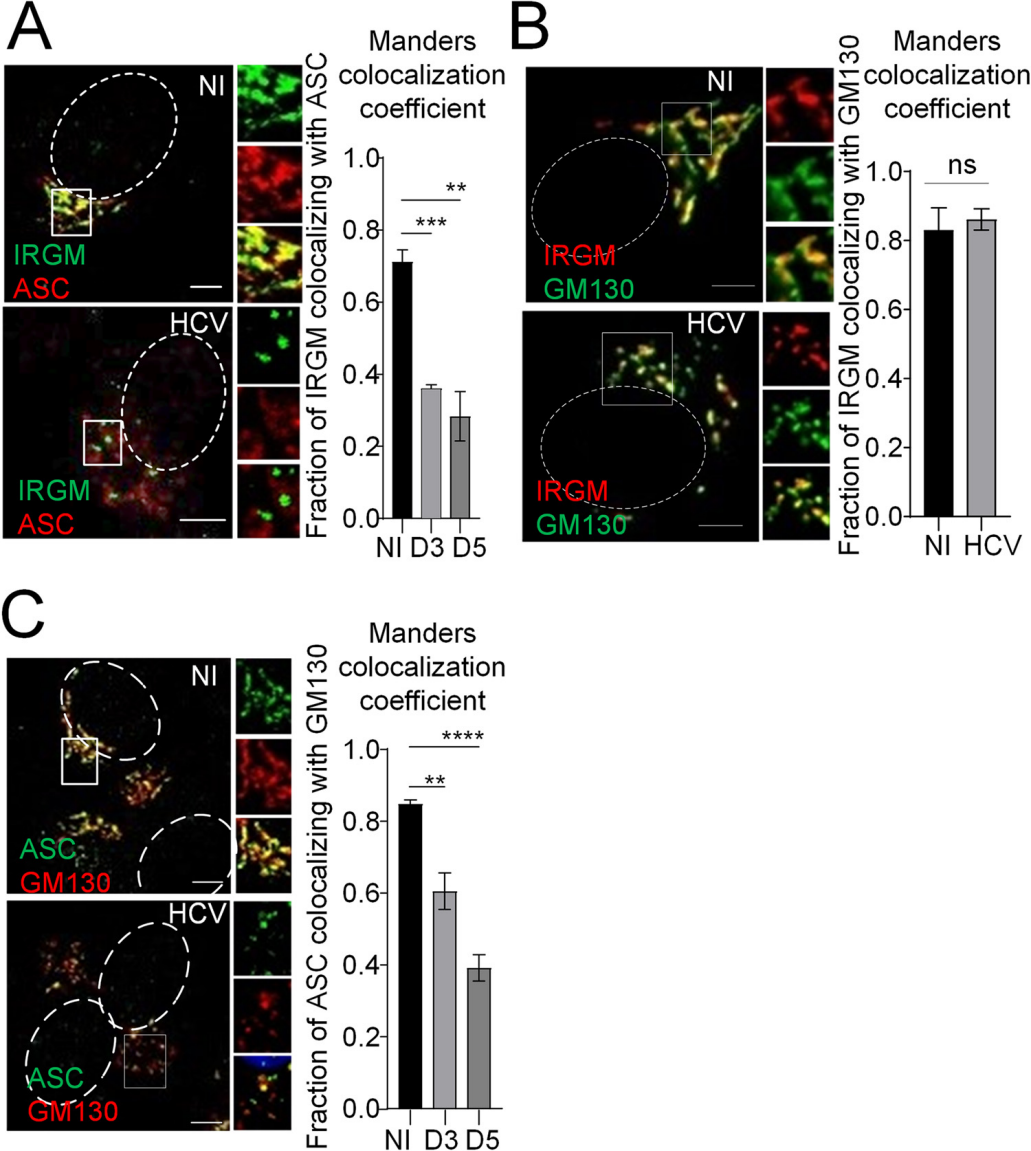
IRGM and ASC colocalize at the Golgi and separate upon HCV infection, together with Golgi fragmentation. (A to C) Huh7 cells were infected or not for the indicated time points. Shown are representative images of cells immunostained with antibodies against (A) IRGM (green) and ASC (red), (B) against IRGM (red) and GM130 (green), or (C) against ASC (green) and GM130 (red). The dotted lines mark the nucleus. The Manders’ colocalization coefficients were calculated using ImageJ. The data shown are means ± SD (*n* = 3 independent experiments). ns, not significant. **, *P* < 0.01; ***, *P* < 0.001; ****, *P* < 0.0001.

**FIG 5:**
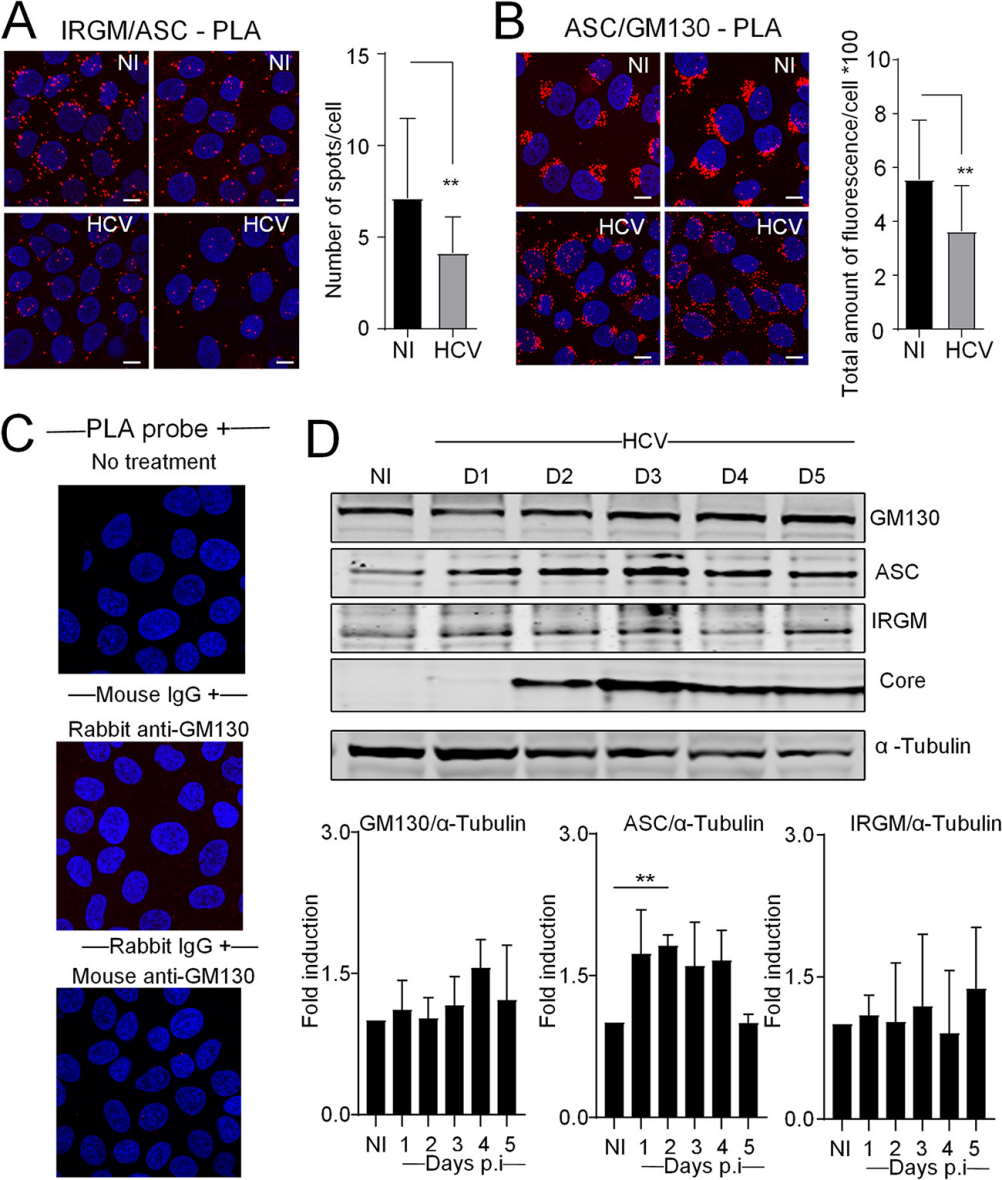
IRGM and ASC colocalize at the Golgi and separate upon HCV infection. (A and B) The proximity ligation assay (PLA) was performed with antibodies directed against ASC and IRGM or against ASC and GM130, with quantification of the number of PLA dots. The data shown are means ± SD (*n* = 3 independent experiments with >180 cells per condition). (C) PLA performed on controls (cells untreated or incubated with each of the antibodies). (D) The kinetics of expression of total protein levels for GM130, ASC, and IRGM together with HCV core protein in uninfected (NI) and HCV-infected Huh7 cells were examined by immunoblotting. α-Tubulin was used as a loading control. A blot from one representative experiment is shown. The data shown are means ± SD (*n* = 3 independent experiments). Scale bar, 5 μm. **, *P* < 0.01.

To assess the possible colocalization of ASC and IRGM in human hepatocytes, we performed confocal microscopy using antibodies against ASC and IRGM. Mander’s coefficient analysis revealed a striking colocalization between ASC and IRGM in resting cells (Fig. 4A, upper panel). IRGM is a Golgi-resident protein (Fig. 4B, upper panel) (11), which might indicate that ASC can also localize at the Golgi. Localization of ASC at the Golgi was then confirmed by its strong colocalization with the *cis*-Golgi marker GM130, assessed by immunofluorescent staining of ASC together with GM130 and by quantification using Mander’s coefficient (Fig. 4C, upper panel). Interestingly, HCV infection impaired both the colocalization of ASC with IRGM (Fig. 4A, lower panel) and that with the Golgi (Fig. 4C, lower panel). Quantification analysis of the IRGM-ASC association using PLA further showed that HCV infection significantly decreases the number of PLA spots per cell (Fig. 5A). This revealed that HCV infection interferes with the IRGM-ASC association. Similarly, quantification analysis of the ASC-GM130 association showed a significant decrease of the frequency of ASC-GM130 PLA spots in the HCV-infected cells compared to uninfected cells (Fig. 5B and controls in Fig. 5C). This shows that HCV infection disrupts, at least in part, the ASC-GM130 association. This stands in contrast to IRGM, which remains associated with Golgi fragments upon HCV infection (Fig. 4B) (11). To rule out that the observed dissociations upon HCV infection might result from lower expression of either protein, we quantified the total protein expression of GM130, ASC, and IRGM over time postinfection with HCV. For GM130 and IRGM, we observed no significant change in the total protein expression, while for ASC, we observed a significant increase in the total protein level during early time points postinfection with HCV (Fig. 5D). Since the protein level of IRGM is not influenced by the HCV infection, this indicates that the reduced fraction of IRGM colocalizing with ASC results from the viral infection and is not a consequence of reduced levels of IRGM protein. The increase in ASC protein levels may influence the fraction of ASC that colocalizes with GM130 to some extent but is probably not the cause of the significant decrease in this colocalization observed in the HCV-infected cells. Also, the decrease is strongest at the time points when ASC levels are comparable to those under the uninfected conditions. Altogether, these results reveal that ASC is a *cis*-Golgi-resident protein, along with IRGM, and that HCV infection triggers its dissociation both from the Golgi apparatus and IRGM.

### ASC is involved in the control of the Golgi structure.

**FIG 6:**
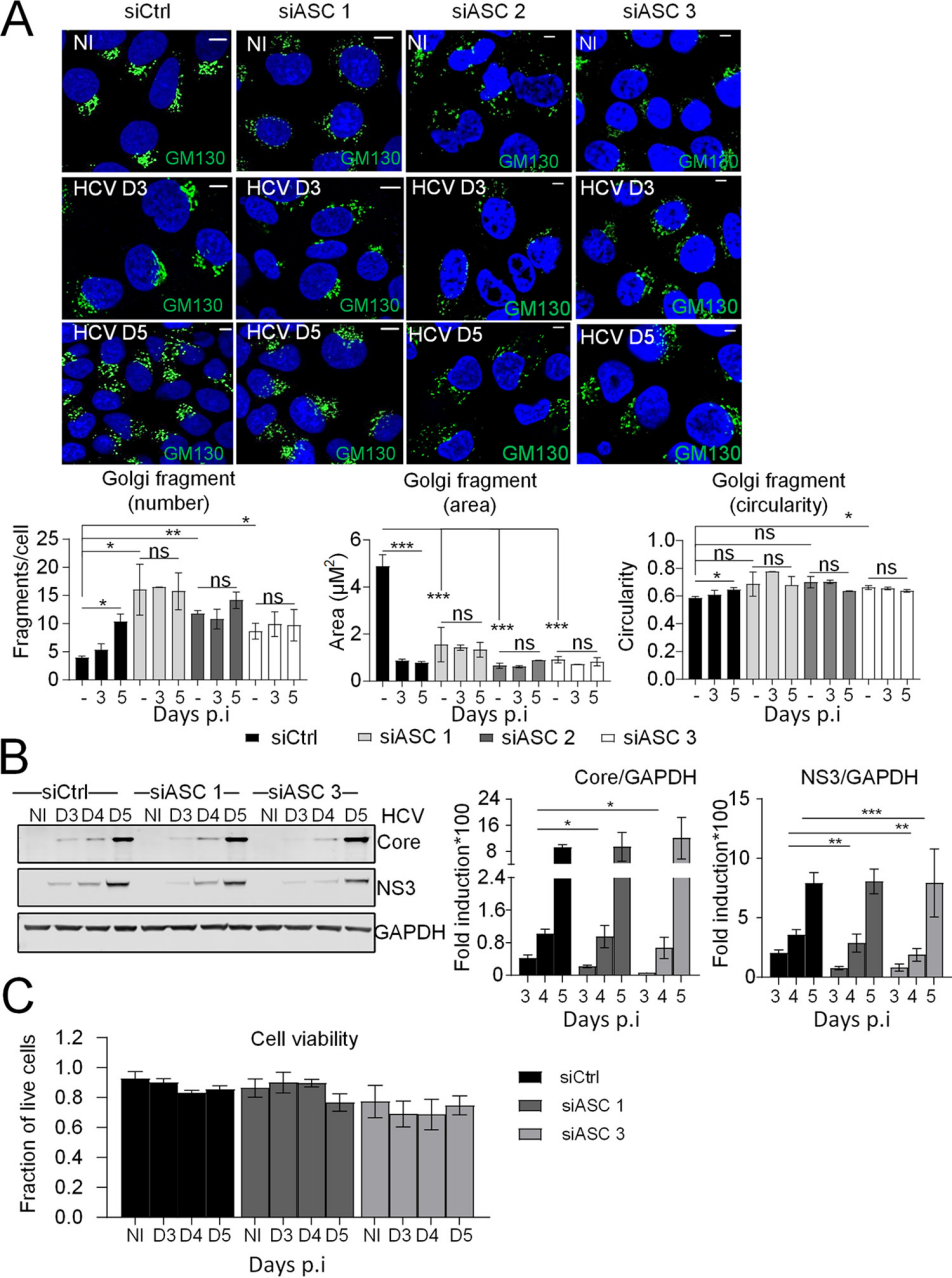
ASC is involved in the control of the Golgi structure. (A) Huh7 cells were transfected with three different siRNAs against ASC and control siRNA prior to infection or not with HCV for 3 and 5 days. Shown are representative confocal images of cells immunostained with antibodies against GM130 (green). Images of cells immunostained for HCV core are not shown. DAPI staining marks nuclei (blue). The characteristics of Golgi fragments were calculated. The data shown are means ± SD (*n* = 3 independent experiments with >50 cells per condition). (B) The effect of ASC depletion on HCV protein levels was examined by immunoblotting using HCV NS3 and HCV core antibodies. Protein levels were normalized to GAPDH. (C) Cell viability was controlled in cells transfected with two of the siRNAs against ASC prior to infection with HCV for 3 and 5 days. Scale bar, 5 μm. ns, not significant. *, *P* < 0.05; **, *P* < 0.01; ***, *P* < 0.001.

We have shown that ASC associates with NLRP3 upon HCV infection (Fig. 1D) and that silencing NLRP3 inhibits Golgi fragmentation in the HCV-infected cells (Fig. 2C). We then examined the effect of silencing ASC on the structure of the Golgi. First, Huh7 cells were transfected with three different siRNAs against ASC and control siRNA as reference prior to their infection or not with HCV for 3 and 5 days. Cells were costained with antibodies against GM130 together with HCV core protein used as a marker to identify HCV-infected cells. To improve readability of the images, the HCV core staining is not shown. Confocal analysis and quantification of the Golgi phenotype revealed that reduction of ASC expression led to a marked increase of Golgi fragmentation, even in uninfected cells, as demonstrated by the higher proportion of small, circular Golgi fragments (Fig. 6A). These data reveal a possible role for ASC in the control of the Golgi structure. In the ASC-silenced cells, HCV was not able to further increase the number of small, circular Golgi fragments already generated by the depletion of ASC (Fig. 6A, lower panels, gray bars), further indicating a role for ASC in the stability of Golgi structure.

Next, we examined the effect of ASC depletion on HCV infection using two of the siRNAs targeting ASC. We found that ASC depletion caused significant reduction of HCV protein levels (HCV core and NS3) at early time points postinfection (Fig. 6B). Of note, no significant changes in cell viability upon ASC depletion were detected (Fig. 6C). Taken together, these results highlight a role for ASC in HCV-induced regulation of Golgi morphology, underlining the importance of Golgi structure integrity in the early time points of HCV infection.

### ASC regulates the localization of IRGM at the Golgi but not its ability to trigger Golgi fragmentation.

**FIG 7:**
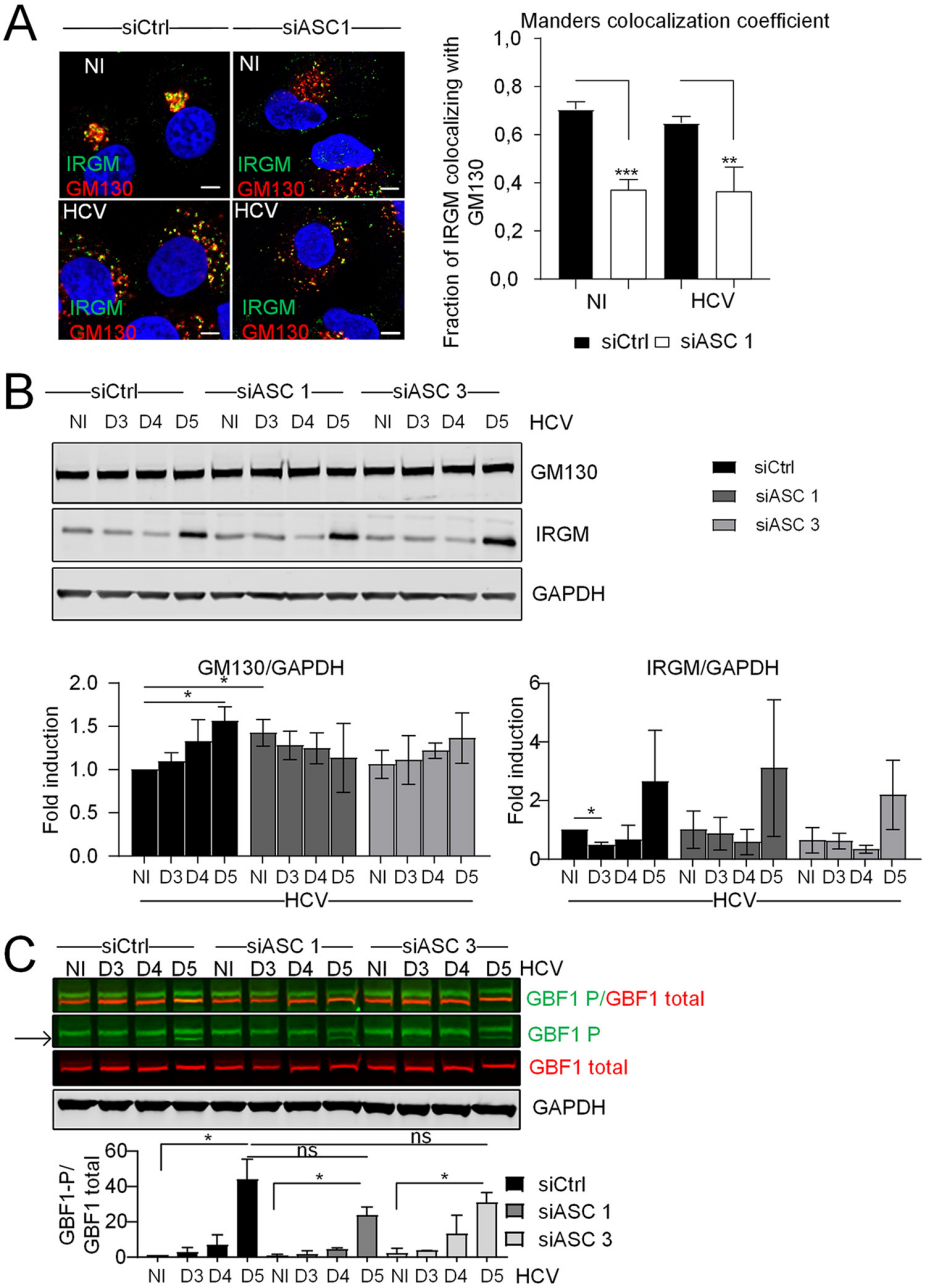
ASC regulates the localization of IRGM at the Golgi but not its ability to trigger Golgi fragmentation. (A) Huh7 cells were transfected with siRNA against ASC (siASC 1) and control siRNA (siCtrl) prior to infection or not with HCV for 5 days. Shown are representative confocal images of cells immunostained with antibodies against IRGM (green) and GM130 (red). DAPI staining marks nuclei (blue). Manders’ colocalization coefficient was calculated. (B and C) Huh7 cells were transfected with two different siRNAs against ASC (siASC 1 and 3) and control siRNA (siCtrl) prior to infection or not with HCV for 3 to 5 days. (B) The effects of ASC depletion on GM130 and IRGM total protein levels were examined by immunoblotting. Protein levels were normalized to GAPDH. (C) The effects of ASC depletion on HCV-induced GBF1 phosphorylation were examined by immunoblotting. A merged image in the first row reveals overlap of signals (seen as orange) between phosphorylated (green) and total GBF1 (red) protein levels. The band corresponding to phospho-GBF1 (GBF1T1337) is indicated by an arrow. GBF1P1337 protein levels were normalized to total GBF1 protein levels. GAPDH was used as a loading control. The data shown are means ± SD (*n* = 3 independent experiments). Scale bar, 5 μm. ns, not significant. *, *P* < 0.05; **, *P* < 0.01; ***, *P* < 0.001.

While ASC appears important to control the structure of the Golgi at homeostasis (Fig. 6), part of it can dissociate from the Golgi and from IRGM upon HCV infection (Fig. 4 and 5). Because of the role of IRGM in Golgi fragmentation during HCV infection (11), we examined the effect of silencing ASC on the localization of IRGM at the Golgi structure in control cells and upon HCV infection. IRGM remained localized with the Golgi apparatus when intact or in HCV-induced Golgi fragments (Fig. 7A; siCtrl), in agreement with our previous report (11). In sharp contrast, ASC silencing greatly reduced the localization of IRGM at the Golgi in both the uninfected and HCV-infected cells (Fig. 7A; siASC). This reduction could not be caused by extensive variations in the total expression levels of either GM130 or IRGM in the uninfected and HCV-infected cells silenced or not for ASC (Fig. 7B). Of note, the increase in the total IRGM protein levels at 5 days postinfection with HCV (Fig. 7B) varied greatly from experiment to experiment and was not considered significant. This result suggests that ASC is involved in the IRGM localization at the *cis*-Golgi (i.e., GM130-positive compartment).

We previously showed that the regulation of Golgi fragmentation by IRGM involved the phosphorylation of the vesicular transport protein GBF1, which is required to attract the Arf1 GTPase at the Golgi and to start the vesiculation process (11). To examine the effect of ASC in this, Huh7 cells were silenced or not for ASC, submitted to the kinetics of HCV infection, and analyzed for the GBF1 phosphorylation state. Our results showed that GBF1 phosphorylation was reduced in the absence of ASC compared to the control cells (Fig. 7C), but the difference was not found to be significant. These data, together with the 50% reduction of IRGM association with the Golgi in the absence of ASC, indicate that the presence of ASC is required for the stability of the association of IRGM with the Golgi, but it is not directly required for the ability of IRGM to mediate GBF1 phosphorylation.

### ASC is involved in the control of the Golgi structure, upstream of IRGM.

**FIG 8:**
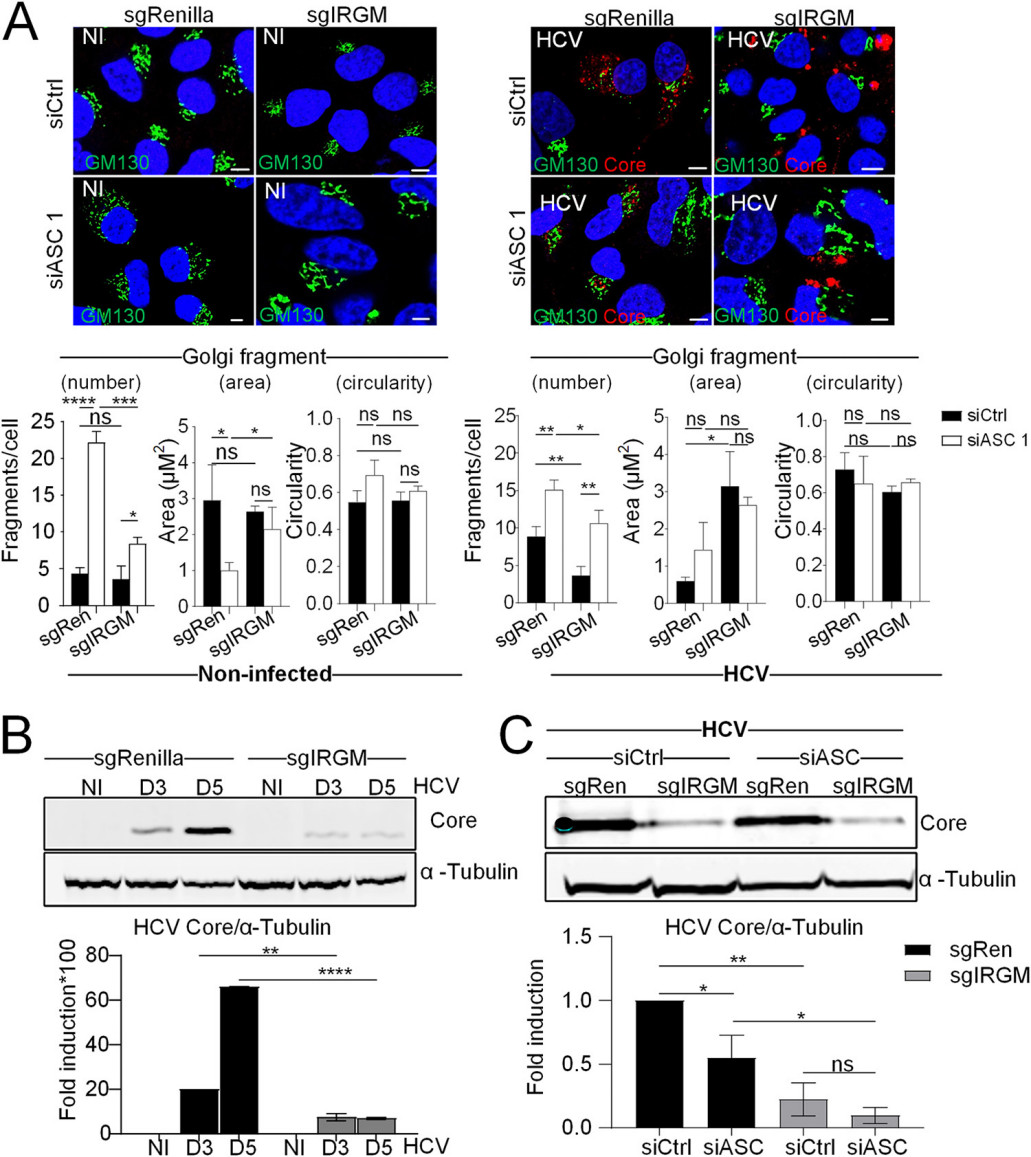
ASC is involved in the control of the Golgi structure, upstream of IRGM. (A) Representative images of CRISPR-modified Huh7 sgIRGM and control sgRenilla cells depleted of ASC or not prior to infection or not with HCV for 3 days and immunostained with antibodies against GM130 (green) and HCV core (red). DAPI staining marks nuclei (blue). The characteristics of Golgi fragments were calculated. The data shown are means ± SD (*n* = 3 independent experiments with >20 cells per condition). (B) The effects of IRGM depletion in CRISPR-modified Huh7 sgIRGM or sgRenilla control cells on HCV core protein levels were examined by immunoblotting and quantified. (C) CRISPR-modified Huh7 sgIRGM and control sgRenilla cells depleted of ASC or not and infected with HCV for 5 days were examined by immunoblotting with an antibody against HCV core protein. The data shown are means ± SD (*n* = 3 independent experiments). Scale bar, 5 μm. ns, not significant. *, *P* < 0.05; **, *P* < 0.01; ***, *P* < 0.001; ****, *P* < 0.0001.

Next, to further determine how ASC intersects with the Golgi structure in the regulation of IRGM, we examined the impact of silencing ASC alone versus combined with IRGM knockout (KO), obtained by the CRISPR/Cas9 approach. Consistently with our previous report (11), IRGM KO does not affect the Golgi structure in the control cells (Fig. 8A; uninfected, black bars) and prevents HCV-induced Golgi fragmentation (Fig. 8A; HCV, black bars). Depletion of ASC caused massive rearrangement of the Golgi structure, generating small, circular vesicles in the single guide RNA *Renilla* (sgRenilla) cells (Fig. 8A; uninfected, white bars), as shown previously for ASC depletion in Huh7 cells (Fig. 6A). In contrast, IRGM KO can only partially prevent Golgi fragmentation resulting from the silencing of ASC. This was observed in both uninfected and HCV-infected cells (Fig. 8A; uninfected and HCV). Next, we confirmed our previous finding (11) that IRGM is a host factor for HCV (Fig. 8B and C). In addition, we found that silencing of ASC has a less inhibitory effect on HCV infection than silencing IRGM, while cosilencing of both IRGM and ASC inhibited HCV infection to a greater extent than either of them alone (Fig. 8C).

Altogether, these data demonstrate a role for ASC in maintaining the Golgi structure, not only at homeostasis (i.e., absence of infection) but also upon HCV infection. This could occur upstream of the ability of IRGM to mediate the HCV-induced Golgi fragmentation, in part via its interaction with IRGM, as well as via possible IRGM-independent regulation.

### Golgi fragmentation in response to nigericin is mediated in part through ASC and IRGM.

**FIG 9:**
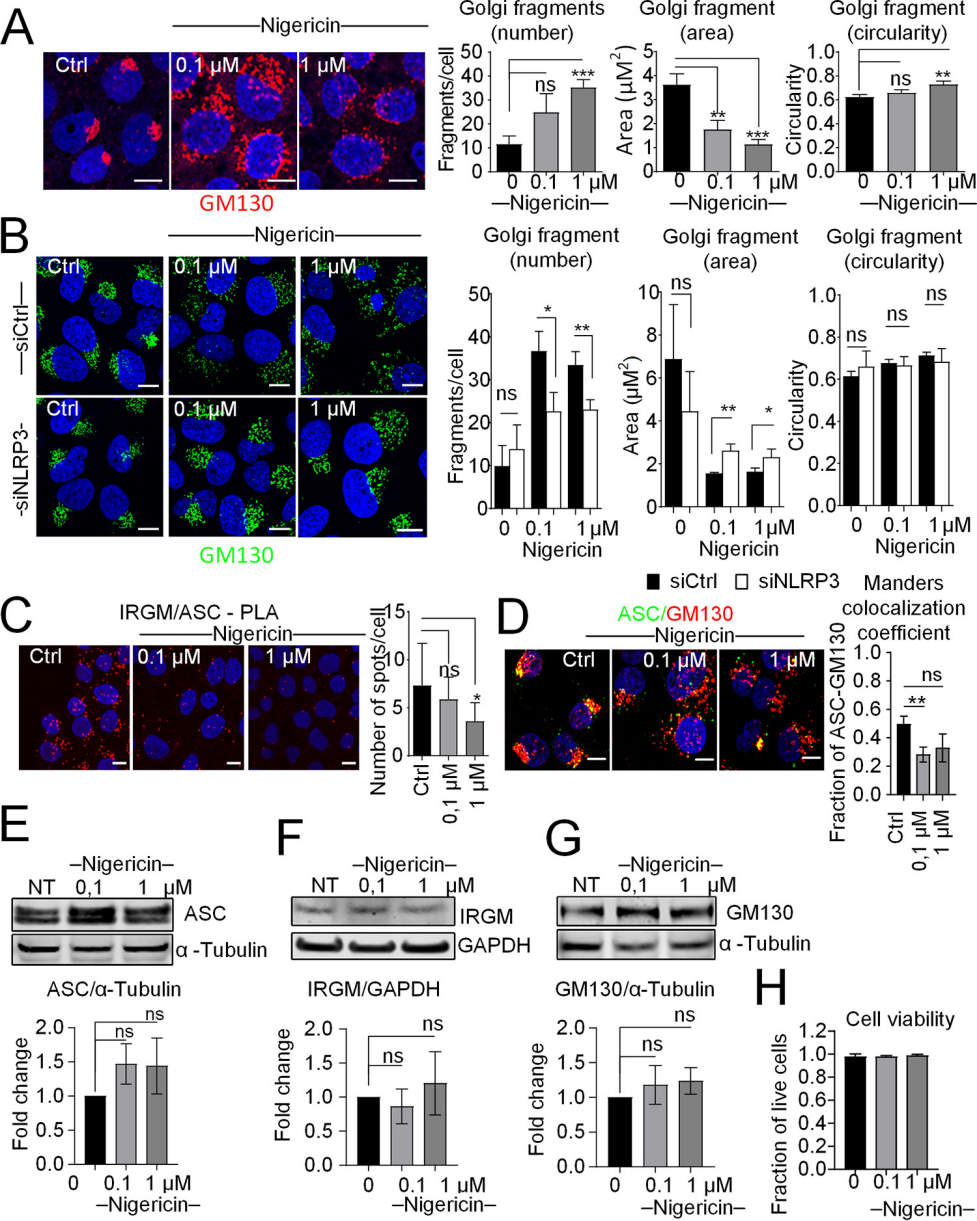
Golgi fragmentation in response to nigericin is mediated in part through ASC and IRGM. (A) Representative images of Huh7 cells not treated (Ctrl) or treated with nigericin (0.1 or 1 μM, 3 h of treatment) prior to immunostaining with an antibody against GM130 (red). DAPI staining marks nuclei (blue). The characteristics of Golgi fragments were calculated. The data shown are means ± SD (*n* = 3 independent experiments with >20 cells per condition). (B) Representative images of cells depleted of NLRP3 or not and not treated (Ctrl) or treated with nigericin (0.1 or 1 μM, 3 h of treatment) prior to immunostaining with antibodies against GM130 (green). DAPI staining marks nuclei (blue). The characteristics of Golgi fragments were calculated. The data shown are means ± SD (*n* = 3 independent experiments with >20 cells per condition). (C) The proximity ligation assay (PLA) was performed in Huh7 cells treated or not with antibodies directed against ASC and IRGM, with quantification of the number of PLA dots. The data shown are means ± SD (*n* = 3 independent experiments with >180 cells per condition). (D) Representative images of cells not treated (Ctrl) or treated with nigericin (0.1 or 1 μM, 3 h of treatment) prior to immunostaining with antibodies against ASC (green) and GM130 (red). DAPI staining marks nuclei (blue). Manders’ colocalization coefficient was calculated using ImageJ. (E to G) Total protein levels of ASC, IRGM, and GM130 were examined in nigericin-treated Huh7 cells and normalized to α-tubulin or GAPDH. Data from a representative experiment for each protein are shown. (H) Cell viability was examined in cells treated with 0.1 and 1 μM nigericin for 3 h or left untreated. The data shown are means ± SD (*n* = 3 independent experiments). Scale bar, 5 μm. ns, not significant. *, *P* < 0.05; **, *P* < 0.01; ***, *P* < 0.001.

Since we demonstrated that NLRP3, ASC, and IRGM are key regulators for the Golgi shape in homeostasis or infection with HCV, we then examined their regulation upon induction of the components of the inflammasome by a virus-unrelated activator, using nigericin (25). Huh7 cells were submitted to nigericin treatment for 3 h. Such time of treatment was sufficient to activate NF-κB, induce NLRP3 expression (Fig. 1F and G), and activate the inflammasome (Fig. 1E). The nigericin-treated or control cells were stained with the *cis*-Golgi marker GM130. Confocal analysis and quantification of Golgi phenotype revealed a clear Golgi fragmentation in response to brief treatment of nigericin, with an increased number of Golgi fragments and reciprocal decreased size, and in a dose-dependent manner (Fig. 9A). Next, confocal analyses and quantification of the Golgi phenotype revealed that the silencing of NLRP3 expression significantly reduced the nigericin-induced rearrangements of Golgi, compared to siCtrl-treated cells (Fig. 9B). These results suggest the existence of a link between NLRP3 and the regulation of Golgi rearrangement, similar to the situation with HCV infection. Then, we showed that nigericin treatment triggers a significant dissociation of ASC from IRGM, as shown by PLA (Fig. 9C), and also from the Golgi, as shown by confocal microscopy using an ASC antibody together with the *cis*-Golgi marker GM130, as quantified by Mander’s colocalization coefficient analysis (Fig. 9D). Of note, nigericin treatment was found to cause no significant changes in the total protein levels of GM130, ASC, or IRGM (Fig. 9E to G) and had no effect of cell viability (Fig. 9H). Together, these results further validate our hypothesis that displacement of ASC from Golgi is associated with Golgi fragmentation.

**FIG 10:**
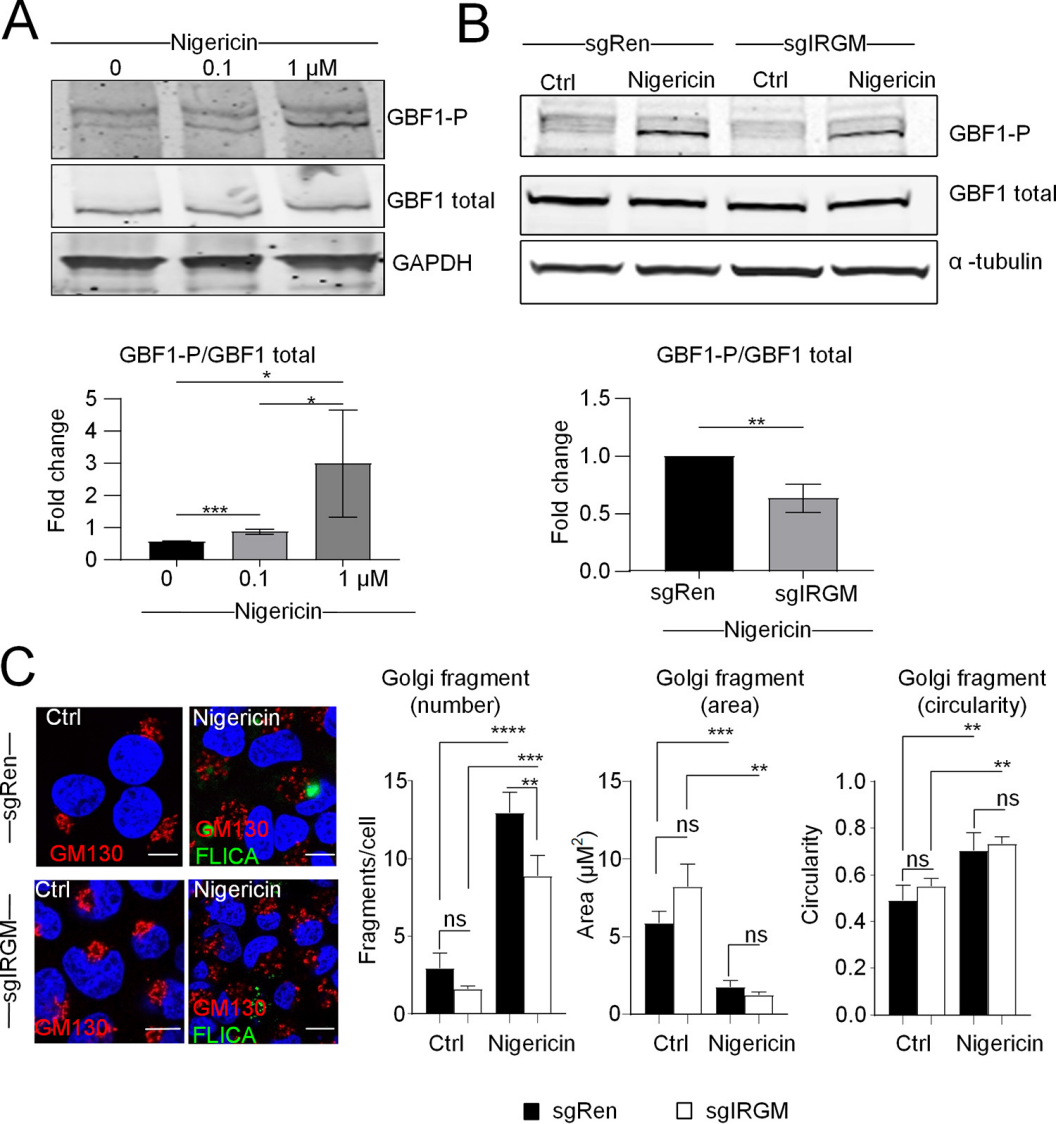
Nigericin mediates GBF1 phosphorylation partly through IRGM. (A) The effect of nigericin on GBF1 phosphorylation was examined by immunoblotting using antibodies directed against the total and phosphorylated (GBF1P1337) forms of GBF1. GAPDH was used as a loading control. GBF1P1337 protein levels were normalized to total GBF1 protein levels. (B) The effect of nigericin (1 μM, 3 h of treatment) on GBF1 phosphorylation was examined in CRISPR-modified Huh7 sgIRGM and control sgRenilla cells by immunoblotting. Ctrl, untreated cells. α-Tubulin was used as a loading control. GBF1P1337 protein levels were normalized to total GBF1 protein levels. (C) Representative images of CRISPR-modified Huh7 sgIRGM and control sgRenilla cells treated or not with nigericin (1 μM, 3 h of treatment). Cells were incubated for 1 h with the FAM-YVAD-FMK reagent (which stains the active form of caspase-1 green) prior to fixation. Fixed cells were immunostained with GM130 (red). DAPI staining marks nuclei (blue). The characteristics of Golgi fragments were calculated. The data shown are means ± SD (*n* = 3 independent experiments with >20 cells per condition). Scale bar, 5 μm. ns, not significant. *, *P* < 0.05; **, *P* < 0.01; ***, *P* < 0.001; ****, *P* < 0.0001.

We then showed that GBF1 phosphorylation was triggered upon nigericin treatment in the Huh7 cells in a dose-dependent manner (Fig. 10A) and was reduced in the IRGM-deficient cells (Fig. 10B). Nonetheless, as opposed to HCV infection, the ability of nigericin to trigger Golgi fragmentation was not totally inhibited by silencing IRGM (Fig. 10C). Altogether, these data indicate that components of the inflammasome, such as NLRP3 and ASC, are involved, at least in part, in the process of the IRGM-mediated Golgi fragmentation, in response to activators other than HCV infection.

### Golgi fragmentation occurs independently of NLRP3 in ZIKV-infected cells.

We then examined whether other viral infections can trigger Golgi fragmentation through NLRP3 and, in particular, whether this occurs in monocytic cell types, in which the inflammasome has been well studied. For this, we used ZIKV, another member of the *Flaviviridae* family, which notably infects monocytes/macrophages (29) thought to be pivotal for viral dissemination. ZIKV has been reported to trigger activation of the NLRP3 inflammasome in monocytes/macrophages (29), and its ability to trigger Golgi fragmentation in neuronal brain cells was observed by electron microscopy (30). Nonetheless, the relationship between the two events is still unknown. Macrophages derived from the monocytic U937 cell line were infected or not with ZIKV, or the cells were treated with nigericin for 3 h as a control. Activation of caspase-1 in response to ZIKV infection was demonstrated using the fluorescent reporter of caspase-1 substrate FAM-FLICA, together with the antibodies recognizing the ZIKV envelop proteins (Env) and thus marking infected cells. For comparison, nigericin treatment for 3 h was used.

**FIG 11:**
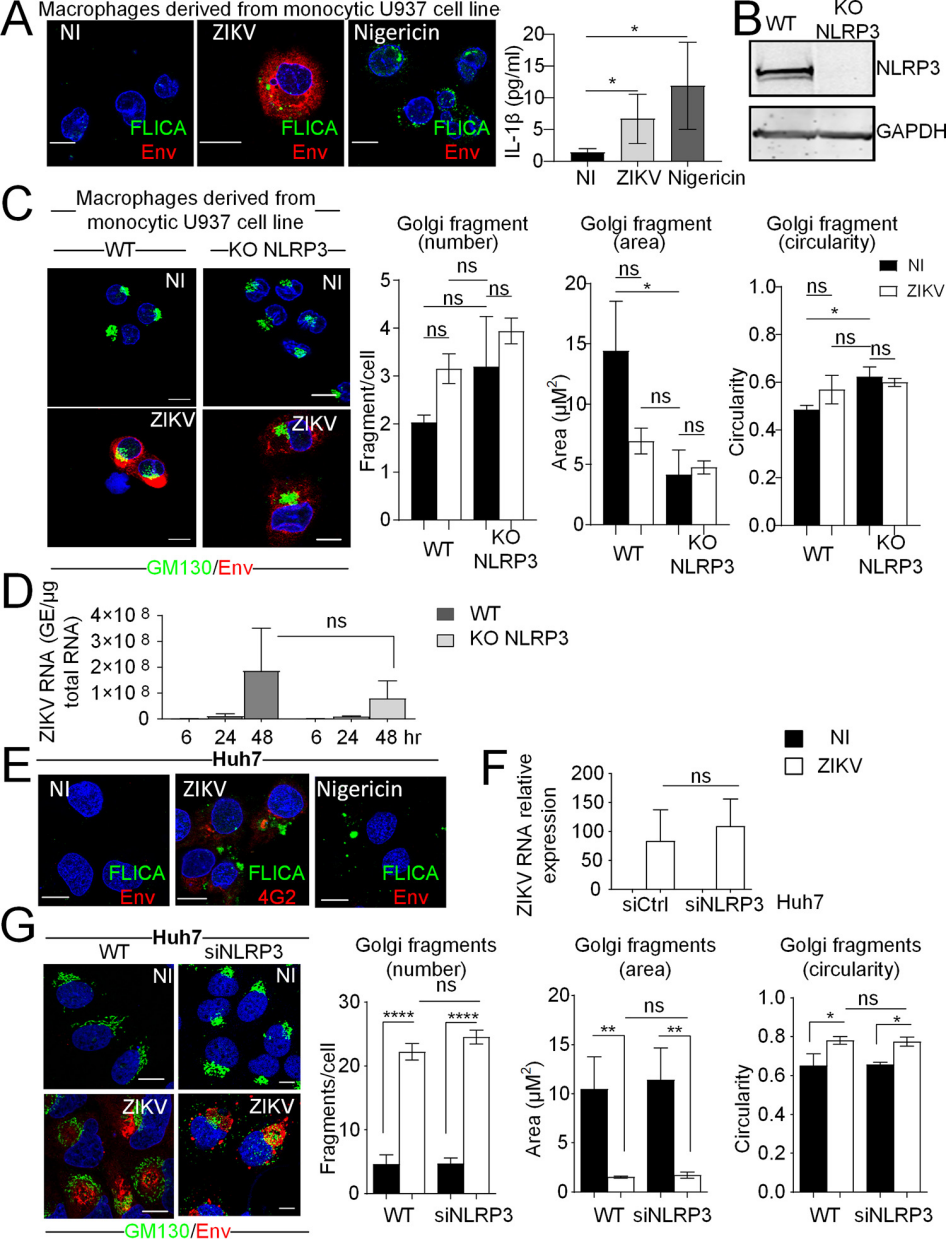
Golgi fragmentation occurs independently of NLRP3 in infection with Zika virus. (A) Macrophages derived from the monocytic U937 cell line were either infected with ZIKV for 48 h or treated with nigericin (1 μM, 3 h of treatment). Control cells were left uninfected and untreated. Cells were incubated for 1 h with the FAM-YVAD-FMK reagent (which stains the active form of caspase-1 green) prior to fixation. Fixed cells were immunostained with antibodies against flavivirus envelope protein (Env; red), and nuclei were stained with DAPI (blue). Secreted IL-1β in ZIKV-infected or nigericin-treated macrophages derived from monocytic U937 cells was examined by ELISA. (B) NLRP3 knockdown in U937 cells was controlled by immunoblotting. GAPDH was used as a loading control. (C) Representative images of macrophages derived from monocytic U937 cells infected for 48 h with ZIKV in WT cells or in NLRP3 KO cells and immunostained with antibodies against GM130 (green) and Env (red). DAPI staining marks nuclei (blue). The characteristics of Golgi fragments were calculated. The data shown are means ± SD (*n* = 3 independent experiments with >20 cells per condition). (D) ZIKV RNA levels were determined by RT-qPCR in the macrophages derived from monocytic U937 cells knocked out for NLRP3 and parental cells infected with ZIKV for the indicated duration. (E) Huh7 cells were either infected with ZIKV for 48 h or treated with nigericin (1 μM, 3 h of treatment). Control cells were left uninfected and untreated. Cells were incubated for 1 h with the FAM-YVAD-FMK reagent (which stains the active form of caspase-1 green) prior to fixation. Fixed cells were immunostained with antibodies against flavivirus envelope protein (Env; red), and nuclei were stained with DAPI (blue). (F) ZIKV RNA levels were determined by RT-qPCR in Huh7 cells transfected with siRNA against NLRP3 or control siRNA infected or not with ZIKV for 48 h. (G) Huh7 cells transfected with siRNA against NLRP3 or control siRNA were infected or not with ZIKV for 48 h. Fixed cells were immunostained with antibodies against flavivirus envelope protein (Env; red), and nuclei were stained with DAPI (blue). The characteristics of Golgi fragments were calculated. The data shown are means ± SD (*n* = 3 independent experiments with >20 cells per condition). Scale bar, 5 μm. ns, *, *P* < 0.05; **, *P* < 0.01; ****, *P* < 0.0001.

We confirmed that the inflammasome was activated in macrophages derived from the monocytic U937 cell line, after 48 h of infection with ZIKV, as shown by activation of caspase-1 (FAM-FLICA labeling in Fig. 11A, left) and secretion of IL-1β (Fig. 11A right). To determine the ability of ZIKV to mediate Golgi fragmentation and evaluate the role of NLRP3 in this, ZIKV infection was carried out in U937 wild-type (WT) and U937 with KO of NLRP3. The absence of NLRP3 expression in the macrophages derived from the monocytic U937 cell line with NLRP3 KO was assessed by immunoblotting (Fig. 11B). We noted an increase, although nonsignificant, in the numbers of Golgi fragments per cell in the U937 NLRP3 KO cells compared to U937 WT cells, in the absence of infection (Fig. 11C; NI, black bars). However, ZIKV infection triggered a small, nonsignificant increase in Golgi fragmentation in the U937 WT cells. The same trend was observed in in the U937 NLRP3 KO cells (Fig. 11C; ZIKV, white bars). This may indicate that ZIKV infection can trigger Golgi fragmentation in macrophages derived from the monocytic U937 cell line, but most probably independently of NLRP3. We then observed that ZIKV viral replication was diminished but not significantly decreased by the reduced NLRP3 expression (Fig. 11D). This may indicate that ZIKV infection may not depend on Golgi-derived lipids in macrophages, in contrast to HCV infection in hepatocytes. To further explore the ZIKV-induced Golgi fragmentation, we performed ZIKV infection in Huh7 cells. First, we confirmed that the inflammasome was activated in ZIKV-infected Huh7 cells by using the fluorescent reporter of caspase-1 substrate (FAM-FLICA), together with an antibody marking ZIKV-infected cells (Env). As a comparison, nigericin treatment for 3 h was used (Fig. 11E). We next observed that ZIKV viral replication was not affected by the reduced NLRP3 expression (Fig. 11F). To determine the ability of ZIKV to mediate Golgi fragmentation and the possible involvement of NLRP3 in this, ZIKV infection was carried out in Huh7 cells depleted or not of NLRP3 by siRNA transfection. We observed a strong induction of Golgi fragmentation induced by ZIKV infection in WT Huh7 cells, but this increase was found to be independent of NLRP3 (Fig. 11G). Altogether, these data indicate that a link between components of the NLRP3 inflammasome and the regulation of the Golgi structure may depend on the nature of the infecting viruses, given their respective requirements from their host.

## DISCUSSION

Here, we have demonstrated that HCV infection triggers IRGM-mediated Golgi fragmentation through NLRP3 and ASC, two proteins that are usually known to be involved in the formation of the inflammasome, and we uncovered a role for ASC in the control of the Golgi structure. We showed that while HCV infection provides all conditions to favor the formation of the NLRP3 inflammasome (induction of NLRP3, interaction of NLRP3 and ASC, and FAM-FLICA labeling), only silencing of NLRP3 inhibits the HCV-mediated Golgi fragmentation, similar to the phenotype we described previously upon IRGM silencing (11). Silencing of ASC did not inhibit Golgi fragmentation and, on the contrary, triggered this fragmentation, even in uninfected cells. This precludes a role for the inflammasome in the HCV-mediated Golgi fragmentation, since this would have been prevented, whether by silencing NLRP3 or ASC. Previous studies on the intracellular localization of ASC were limited and somewhat contradictory, placing ASC in the cytosol, at the mitochondria, or in the nucleus (26–28). Using different siRNAs against ASC to assess the specificity of our anti-ASC antibodies, we unambiguously showed that ASC resides at the Golgi under homeostatic conditions, in association with the *cis*-Golgi-resident IRGM and GM130. HCV infection triggers an increased association between ASC and the HCV-induced NLRP3 and the dissociation of ASC from the Golgi and from IRGM, while IRGM still localizes to the Golgi. The mechanisms leading to the formation of the NLRP3 inflammasome upon HCV infection are not known yet, but could be related to modifications of NLRP3, such as its phosphorylation by Jun N-terminal protein kinase (JNK1) (31, 32) or protein kinase D (PKD) (33, 34). Here, we did not address the phosphorylation change in NLRP3 upon HCV infection, but we showed that its expression is induced upon HCV infection, that it associates with ASC, and importantly, that its depletion by silencing prevents Golgi fragmentation. Our data imply a role for NLRP3 in HCV infection and highlight a role of ASC at the Golgi.

**FIG 12:**
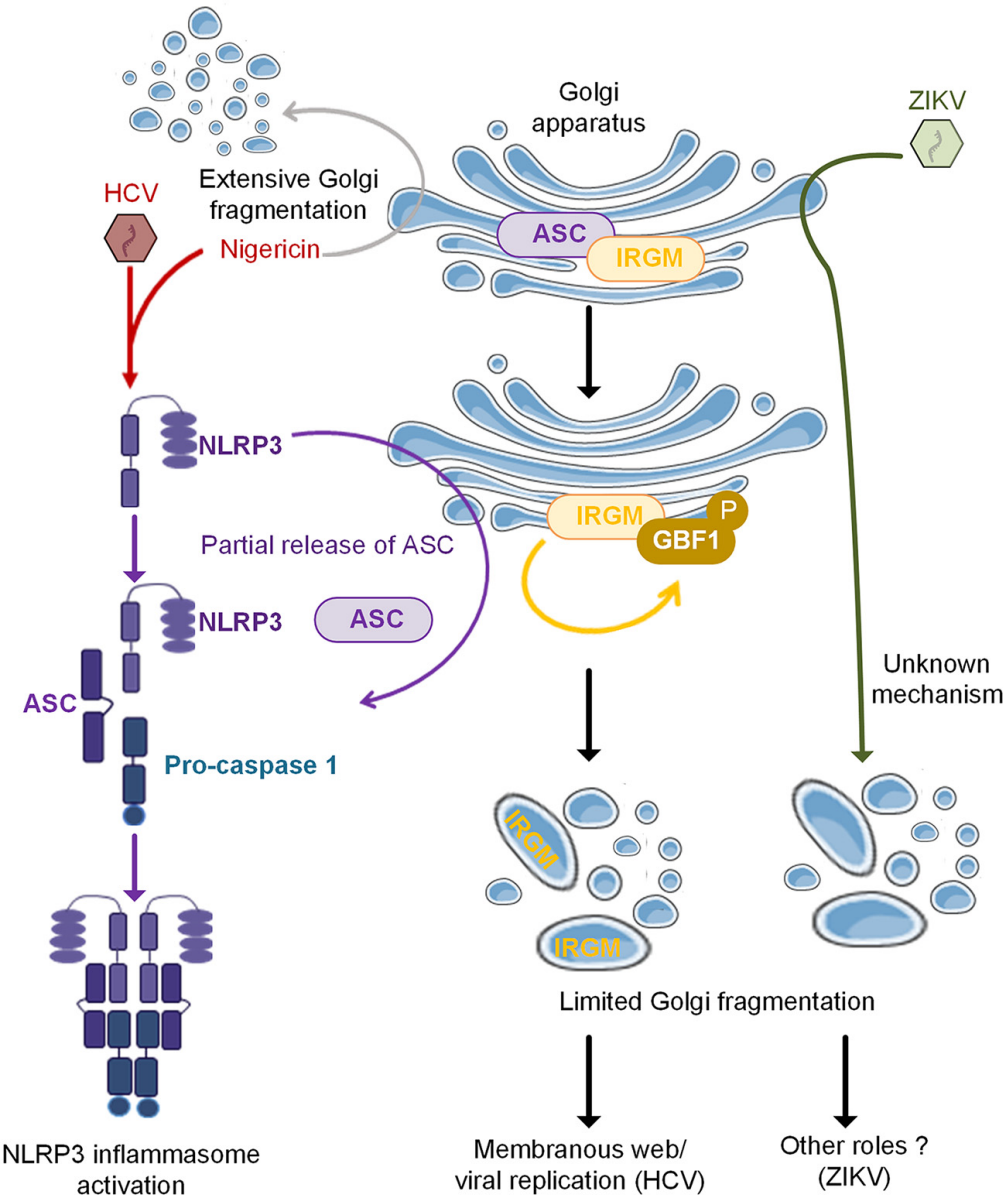
Proposed model for the role of NLRP3 and ASC in the IRGM-mediated Golgi fragmentation. Based on our results, we show that ASC is a Golgi-resident protein at homeostasis, where it colocalizes with IRGM. HCV infection triggers induction of NLRP3, and we propose that this leads to a partial release of ASC from the Golgi, due to its association with NLRP3 in view of forming an active NLRP3 inflammasome. Such release of ASC from the Golgi “liberates” IRGM, which can then trigger Golgi fragmentation through GBF1 phosphorylation, as reported previously (11). A link between Golgi fragmentation and NLRP3 can also occur after treatment with the potassium ionophore nigericin. An NLRP3-independent extensive Golgi fragmentation was also noted in response to nigericin. In contrast to HCV, ZIKV induces Golgi fragmentation, but in an NLRP3-independent manner, and viral replication is not significantly decreased upon NLRP3 depletion. The role of Golgi fragmentation during ZIKV infection remains to be determined.

Upon HCV infection, dissociation of ASC from the Golgi may consequently lead to its dissociation from IRGM and favor the ability of IRGM to trigger GBF1 phosphorylation and formation of Golgi vesicles (Fig. 12). Since the intact Golgi structure was only partially restored by silencing IRGM in control cells silenced for ASC, this demonstrates that the role of ASC is not to directly regulate IRGM function through its association but rather to control the maintenance of the Golgi structure in absence of inflammasome activation. We hypothesize that in response to HCV infection, only a fraction of the ASC population is released from IRGM (and probably from other partners) to associate with NLRP3 to form the inflammasome. Consequently, IRGM would remain associated with sufficiently organized Golgi structures to be able to mediate some Golgi fragmentation through GBF1 phosphorylation. We also showed that NLRP3 was involved in Golgi fragmentation upon a short treatment with nigericin. In contrast, we observed that, while ZIKV infection could trigger Golgi fragmentation in macrophages derived from both monocytic U937 cells and Huh7 cells, this was NLRP3 independent, unlike HCV. Since NLRP3 depletion does not prevent the Golgi fragmentation induced by ZIKV in either of the cell lines used, this implies that other mechanisms/pathways can be in play in the context of ZIKV infection (Fig. 12). It is possible that this may be related to different viral replication strategies used by the two viruses belonging to the same family and the fact that ZIKV infection may not need the Golgi-derived supply of lipids similar to HCV, as its replication process is more embedded within the ER (35). Alternatively, ZIKV may use Golgi fragmentation to favor its secretion (36). Altogether, our results highlight virus-induced changes in Golgi morphology in both nonhematopoietic and hematopoietic cells and a role for inflammasome components in these processes during HCV infection and treatment with nigericin (Fig. 12).

The Golgi complex is organized in tubular structures in association with microtubules at a perinuclear localization. Although it is commonly represented as a crescent-like twisted ribbon structure, the Golgi apparatus is now recognized as a very dynamic structure, which can adopt different configurations depending on the state of the cells (37). This structure is maintained through the action of several Golgi-resident proteins, such as GBF1 (38) and the GRASP proteins (Golgi reassembly and stacking proteins) (39, 40). Alteration of this structure can occur in different situations, resulting notably in Golgi fragmentation. For instance, during the G_2_ phase of mitosis, JNK2 phosphorylates GRASP65, a protein that links the different Golgi cisternae, conveniently leading to equal transfer of the isolated Golgi stacks to the daughter cells (39). GBF1, a member of the guanine exchange factor (GEF) family, maintains the integrity of the Golgi and coordinates the bidirectional transport of the COP proteins through the small GTPase Arf1. Phosphorylation of GBF1 allows the binding of Arf1 under its GTP-binding form to the Golgi, resulting in Golgi fragmentation. The modulation of GBF1 activity is observed during several infections, such as those caused by the enteroviruses poliovirus and coxsackievirus B3, for which GBF1 is crucial for viral replication (41). Recently, we showed that HCV triggers GBF1 phosphorylation and subsequent Golgi fragmentation through IRGM (11). This process also benefits HCV replication, as Golgi vesicular membranes can migrate to the viral replication complex, where they can be used as lipid supply.

Very recent work suggested key functions for the Golgi apparatus and the ER-Golgi vesicle trafficking in the assembly of the NLRP3 inflammasome (42–44). Moreover, disruption of Golgi integrity may inhibit NLRP3 inflammasome activation or, on the contrary, may serve as a scaffold for NLRP3 aggregation and activation (42). This suggests that mediators from these organelles may contribute to inflammasome assembly.

Our data highlight that inflammasome components are utilized by HCV in order to generate Golgi-derived lipids as a supply for viral replication and assembly. HCV triggers the formation of a so-called membranous web close to the ER (5, 6). The formation of these structures requires the presence of lipids, which can be generated on site or delivered through transportation, such as that provided through Golgi fragmentation. Thus, induction of the NLRP3 by HCV benefits the virus as it can lead to destabilization of the Golgi structure, thus allowing IRGM to further ensure Golgi fragmentation, allowing a supply of Golgi-derived lipids for HCV replication.

While this work was in progress, IRGM was reported to interact with ASC (45). Interestingly, in this study, the IRGM-ASC interaction was observed in the inflammasome specks in hematopoietic cells submitted to different microbial PAMPs (pathogen-associated molecular patterns), and IRGM was interrupting the interaction between NLRP3 and ASC, thus negatively controlling the NLRP3 inflammasome. On the other hand, we showed that IRGM and ASC interact at the Golgi under homeostatic conditions and that they separate upon the presence and presumably activation of NLRP3. Thus, it is possible that the interaction of ASC and IRGM is essential to control the activity of each of these proteins. ASC retains IRGM at the Golgi and prevents its ability to trigger Golgi fragmentation, while the association of IRGM with the inflammasome specks exerts a feedback control on the inflammasome by interrupting the NLRP3-ASC interaction. In line with this, the depletion of CPTP (ceramide-1-phosphate transfer protein), involved in the traffic of ceramide-1-phosphate from the *trans*-Golgi network to membranes, triggers Golgi fragmentation and activation of the NLRP3 inflammasome (46). Future investigation will be needed to determine the effect of silencing of IRGM on Golgi structure and the inflammasome in this context.

IRGM was previously identified as a risk factor for Crohn’s disease and tuberculosis and for its role in autophagy. Data from Mehto et al. (45) provided a mechanistic explanation for the ability of IRGM to control inflammasome assembly through its interaction with ASC at the NLRP3 inflammasome and dragging the inflammasome to autophagosomes. Reciprocally, our data now provide a mechanistic explanation for the ability of the NLRP3 and ASC components of the inflammasome to trigger the IRGM-mediated Golgi fragmentation and autophagy. Golgi structure is known to be altered by stress related to disease (47). In particular, a dispersed Golgi phenotype has been observed in several cancer cell lines and may play a central role in metastasis (48–51). Additionally, in Alzheimer’s disease, the Golgi is known to undergo fragmentation, and it is thought that fortifying Golgi stability could be a potential therapeutic strategy (52–54). Here, we uncovered that Golgi fragmentation, under conditions other than mitosis and cell division, can be considered an important regulatory aspect of several pathological situations arising from some viral infections. It should be therefore of great interest to examine the state of IRGM polymorphism in some chronic infections.

## MATERIALS AND METHODS

### Cell culture and virus.

Cells of the hepatoma cell line Huh7 (55) were maintained in Dulbecco’s modified Eagle’s medium (DMEM) supplemented with 10% fetal bovine serum (FBS) (F7424; Sigma), 1% penicillin-streptomycin (Life Technologies), and 1% nonessential amino acids (Sigma) (thereafter referred as complete medium) and cultured at 37°C in 5% CO_2_ The Huh7.25/CD81 and the Huh7.25/CD81/sgIRGM or Huh7.25/CD81/sgRenilla clones (11) were maintained in the same medium with addition of 0.4 mg/ml Geneticin (G418) for all cells and 10 μg/ml puromycin for the two latter ones. Macrophages were derived from monocytic U937 cells, which were knocked out (KO) or not (parental WT) for NLRP3. U937 cells (obtained from Centre de Ressources Biologiques, CelluloNet) were genetically modified by the CRISPR/cas9 approach for the invalidation of NLRP3, as previously described (56). U937 cells and derivates were cultured in RPMI 1640 supplemented with 10% FBS, 100 IU/ml penicillin, and 100 μg/ml streptomycin (all from Life Technologies) at 37°C in 5% CO_2_ and differentiated into macrophage-like phenotype cells by adding 100 nM phorbol 12-myristate 13-acetate (PMA; InvivoGen) 2 days prior to infection by ZIKV.

JFH1 HCV stocks were produced in Huh7.25/CD81 cells and titrated as described previously (55). HCV infections were performed at a multiplicity of infection (MOI) of 0.3 in complete medium for 2 h before the removal of virus and incubation for the indicated time points. For 5 days of infection, cells were trypsinized and split at day 3 postinfection before further cell expansion for the 2 remaining days.

The Brazilian clinical isolate of (ZIKV, PE243_KX197192) (57), and the low-passage-number PF-25013-18 strain of ZIKV (58) were amplified in Vero cells. The two ZIKV strains belong to the Asian lineage (59). The supernatant was collected 3 days postinfection, filtered with 0.45-μm-pore filter (Corning), and stored at −80°C. Prior to infection of macrophages from monocytic U937 NLRP3 KO cells and parental cells or Huh7 cells, ZIKV was incubated with 4G2 antibody (1:100 dilution; hybridoma supernatant-containing anti-Env glycoprotein of flaviviruses) for 1 h at 37°C. ZIKV infections were performed at an MOI of 5 or 10 for U937 and Huh7 cells at the indicated time points.

### Reagents and antibodies.

Primary antibodies were purchased from the following suppliers: from Santa Cruz Biotechnology, HCV core mouse monoclonal antibody (Mab) sc-57800 and ASC mouse MAb sc-514414; from Cell Signaling Technologies, NLRP3 rabbit MAb 15101S and normal rabbit IgG 2729; from Abcam, IRGM mouse MAb ab-69494 and GM130 rabbit MAb 52649; from Sigma, GAPDH (glyceraldehyde-3-phosphate dehydrogenase) mouse MAb g8795; from BD Transduction Laboratories, GM130 mouse MAb 610822 and total GBF1 mouse MAb 612116; from IBL, P-GBF1 (T1337 phosphorylated) rabbit Ab 28065; and from Adipogen, NLRP3 mouse MAb AG-20B-0014 and ASC rabbit Ab AG-25B-0006. The hybridoma-producing antibodies against flavivirus envelope protein 4G2 were kindly provided by P. Despres (PIMIT, Université de La Réunion-INSERM France). Secondary antibodies for immunoblotting were from Thermo Scientific: goat anti-mouse IgG conjugated with DyLight 800 and DyLight 680 and goat anti-rabbit IgG conjugated with DyLight 800 and DyLight 680. Secondary antibodies for immunofluorescence were from Invitrogen: Alexa Fluor 488-conjugated goat anti-mouse and goat anti-rabbit and Alexa Fluor 568-conjugated goat anti-mouse and goat anti-rabbit. Antiprotease cOmplete and antiphosphatase PhoSTOP were from Roche. Nigericin was from Sigma. Caspase-1 activation was detected using the FAM-FLICA Caspase1 assay kit (ImmunoChemistry Technologies). Reagents and antibodies were used following the manufacturer’s instructions.

### RNAi.

The following siRNA oligonucleotides were used for RNA interference (RNAi): NLRP3, 114548 (Dharmacon); ASC, 1027416 (Qiagen); AM51331 (Ambion), siRNA 44415 (siASC 2); 4392420 (Ambion), siRNA s26508 (siASC 3); and negative-control siRNA D-001810-10-20 (Dharmacon). siRNA duplexes were reverse transfected into cells using Lipofectamine RNAiMAX (17-0618-0; Invitrogen) on the day of plating (24 h before infection or treatment) according to the manufacturer’s instructions. Two subsequent transfections were performed on adherent cells: the first one before infection and the second one on day 3 postinfection.

### Quantitative real-time PCR analysis.

Total cellular RNA was extracted using TRI Reagent (Sigma), according to the manufacturer’s instructions. cDNA was generated from 1 μg of total RNA using the Revert Aid reagents (Thermo Scientific). The resulting cDNA was diluted 1:20 and subjected to semiquantitative real-time PCR (RT-qPCR) using the SYBR green reagent (Life Technologies) and specific primers. Those primers were as follows: JFH1, 5′-TCCCGGGAGAGCCATAGTG-3′ (forward) and 5′-TCCAGGAAAGGACCCAGTC-3′ (reverse); Roth, 5′-CTGCGGACTATCTCTCCCCTC-3′ (forward) and 5′-AAAAGGCTTTGCAGCTCCAC-3′ (reverse); ZIKV, 5′-ATTGTTGGTGCAACACGACG-3′ (forward) and 5′-CCTAGTGGAATGGGAGGGGA-3′ (reverse); and IL-8, 5′-AAGGGCCAAGAGAATATCCGAA-3′ (forward) and 5′-ACTAGGGTTGCCAGATTTAACA-3′ (reverse). Amplification reactions were performed under the following conditions: 2 min at 50°C, 10 min at 95°C, and 40 cycles of 10 s at 95°C and 1 min at 60°C. As specified by the manufacturer, relative transcript levels were calculated by the threshold cycle (Δ*C_T_*) method, using Roth as the reference gene.

### Immunofluorescence.

Cells were fixed in phosphate-buffered saline (PBS) containing 4% paraformaldehyde (PFA) for 15 min on ice and were permeabilized, and nonspecific antibody sites were blocked with PBS containing 10% FBS, 2.5% bovine serum albumin (BSA), and 0.3% Triton X-100 for 30 min at room temperature. For staining, cells were incubated with antibodies diluted in blocking solution. For U937 cells, human Fc blocking reagent (MACS; Miltenyi Biotec) was added to this blocking step. Primary antibodies were added for 60 min at 37°C or at 4°C overnight. Secondary antibodies were added for 30 min at room temperature. Nuclei were visualized by incubation with DAPI (4′,6-diamidino-2-phenylindole) for 5 min at room temperature. For staining of IRGM proteins, cells were fixed and permeabilized with 100% ice-cold methanol at −20°C for 10 min and blocked with PBS containing 10% FBS and 2.5% BSA for 30 min at room temperature.

### Confocal fluorescence microscopy.

Confocal fluorescence microscopy studies were performed with a Zeiss Axiovert 200-M inverted microscope equipped with an LSM 510 or 700 laser-scanning unit and a 1.4 NA, 63× Plan-Apochromat oil-immersion objective. Cells were seeded in μ-Slide eight-well, ibiTreat tissue culture-treated plates (Inter Instrument AS), in 12-well plates containing glass coverslips (Sigma), or in 96-well glass bottom plates (Nunc). The glass coverslips were mounted with Prolong (Invitrogen). To minimize photobleaching, the laser power typically was 20% under maximum and the pinhole was set to 0.8 to 1.5. Multitracking was used for dual- or triple-color imaging. Quantitative confocal image analysis was performed using ImageJ software. The fluorescence intensity of Golgi objects (based on GM130 staining) was determined by background subtraction and using a fixed threshold. Otsu’s thresholding algorithm was applied to convert images to binary images and to create regions of interest (ROIs) based on pixel intensity. Then the particle area and circularity were quantified using the Analyze Particle plugin in ImageJ (https://imagej.nih.gov/ij). A circularity value of 1 corresponds to a perfect circle (60). Colocalization analysis was performed using the JaCoP plugin in ImageJ (61) Image parameters remained constant during imaging, and threshold values were kept the same from image to image during image analysis. Mander’s colocalization coefficient (MCC) was used. MCCs measure the fraction of one protein that colocalizes with a second protein (62).

### Proximity ligation assay.

Cells were cultured on 24-well plates containing glass coverslips, washed with PBS, and fixed and permeabilized with acetone and methanol (isovolume) for 5 min at −20°C, except for the ASC-NLRP3 proximity ligation assay (PLA), where the cells were fixed with 4% paraformaldehyde (PFA) for 15 min at room temperature and permeabilized with 0.01% digitonin for 30 min at room temperature.

The proximity ligation assay was performed using the DuoLink PLA technology (Sigma). Briefly, the cells were incubated with Duolink blocking solution for 1 h at 37°C in a preheated humidity chamber and then with primary antibodies overnight at 4°C. The coverslips were washed with buffer A and incubated with the plus and minus PLA probes (antibodies with attached oligonucleotide strands) for 1 h at 37°C. After three washes with buffer A, the PLA probes were hybridized to connect oligonucleotides, which were then joined in a ligation step for 30 min at 37°C. After three more washes with buffer A, the resulting closed circular DNA template was amplified by DNA polymerase for 100 min at 37°C. Oligonucleotides coupled to fluorochromes contained in the amplification buffer hybridized to repeated motifs on the amplicons, allowing detection. Coverslips were then washed with buffer B and mounted with Duolink *in situ* mounting medium containing DAPI. Images were collected (Zeiss Inverted LSM 700 Microscope) and analyzed with ImageJ: a projection of Z-stack PLA blobs was merged with nuclei labeled with DAPI.

### Immunoblotting.

Cells were washed once with PBS and lysed either in CHAPS {3-[(3-cholamidopropyl)-dimethylammonio]-1-propanesulfonate} lysis buffer containing 50 mM Tris (pH 7.5), 140 mM NaCl, 5% glycerol, 1% CHAPS, 2 mM EDTA, and 40 mM glycerophosphate or in 8 M urea buffer, both containing protease and phosphatase inhibitors (Roche cOmplete inhibitor cocktail). Cell lysates were clarified by centrifugation, separated by lithium dodecyl sulfate (LDS)-PAGE, and electrophoretically transferred to nitrocellulose membranes using the iBlot transfer system (Invitrogen, Life Technologies). Membranes were blocked in Li-Cor Odyssey blocking buffer at a 1:1 ratio with Tris-buffered saline (TBS) for 1 h at room temperature before incubation with the indicated antibodies overnight at 4°C. After washes with TBS containing 0.1% Tween 20, immunoreactive proteins were detected with Li-COR DyLight secondary antibodies and visualized on the Li-COR Odyssey system.

### ELISA.

The amount of IL-1β in cell-culture supernatants was quantified by enzyme-linked immunosorbent assay (ELISA) using the IL-1β/IL-1-F2 kit (DY 201; R&D Systems), according to the manufacturer’s instructions. The detection limit was 1 pg/ml.

### Cell viability assay.

Cells were washed once with ice-cold PBS and trypsinized for 5 min at 37°C. The cell suspension was diluted 1:1 in Trypan blue, and the ratio of live to dead cells (percentage) was calculated using a Bio-Rad TC20 cell counter.

### Statistical analysis.

For quantification of immunofluorescence microscopy images, a minimum of 20 cells were counted for each condition in each experiment. Three independent experiments were performed for all figures. Data are shown as means ± standard deviation (SD). *P* values were calculated by using paired Student *t* test when we compared two conditions. In experiments where we compared multiple groups (e.g., comparison of uninfected to infected cells that received different treatments or at different time points), we used two-way analysis of variance (ANOVA) with Tukey’s multiple-comparison test. A *P* value of <0.05 is considered statistically significant.
